# The genome sequence of a marine yeast, *Metschnikowia zobellii *(Uden & Cast.-Branco, 1961)

**DOI:** 10.12688/wellcomeopenres.19998.1

**Published:** 2023-09-19

**Authors:** Michael Cunliffe, Ro Allen, Nathan Chrismas, Marine Biological Association Genome Acquisition Lab, Darwin Tree of Life Barcoding collective, Wellcome Sanger Institute Tree of Life programme, Wellcome Sanger Institute Scientific Operations: DNA Pipelines collective, Tree of Life Core Informatics collective, Darwin Tree of Life Consortium

**Affiliations:** 1 The Marine Biological Association, Plymouth, England, UK

**Keywords:** Metschnikowia zobellii, marine yeast, genome sequence, chromosomal, Saccharomycetales

## Abstract

We present a genome assembly from a *Metschnikowia zobellii culture* (a marine yeast; Ascomycota; Saccharomycetes; Saccharomycetales; Metschnikowiaceae). The genome sequence is 13.6 megabases in span. Most of the assembly is scaffolded into 5 chromosomal pseudomolecules. The mitochondrial genome has also been assembled and is 51.12 kilobases in length.

## Species taxonomy

Eukaryota; Opisthokonta; Fungi; Dikarya; Ascomycota; saccharomyceta; Saccharomycotina; Saccharomycetes; Saccharomycetales; CUG-Ser1 clade; Metschnikowiaceae; *Metschnikowia*; *Metschnikowia zobellii* ( Uden & Cast.-Branco, 1961) (NCBI:txid27328).

## Background

*Metschnikowia zobellii* ( van Uden & Castelo-Branco, 1961) van Uden, 1962 is a marine ascomycete yeast in the family Metschnikowiaceae which infects small crustaceans, including copepods ( Seki & Fulton, 1969). *M. zobellii* spores are elongated and barbed, with one spore occurring per ascus ( Mendonca-Hagler *et al.*, 1993). These needle-like spores penetrate the gut walls of grazing invertebrates where the yeast multiples, eventually killing the host ( van Uden & Castelo-Branco, 1961). *Metschnikowia* are components of coastal marine planktonic communities that reoccur year on year ( Chrismas *et al.*, 2023) and may play an important role in plankton population dynamics.

The genome of *M. zobellii* was sequenced as part of the Darwin Tree of Life Project, a collaborative effort to sequence all named eukaryotic species in the Atlantic Archipelago of Britain and Ireland. This genome is one of the first marine fungal genomes available to the scientific community and will provide new insights and opportunities for marine fungal research.

## Genome sequence report

The genome was sequenced from a colony of *Metschnikowia zobellii* collected from Church Reef, Wembury, Devon, UK (50.32, –4.08). A total of 90-fold coverage in Pacific Biosciences single-molecule HiFi long reads was generated. Primary assembly contigs were scaffolded with chromosome conformation Hi-C data.

**Figure 1.**
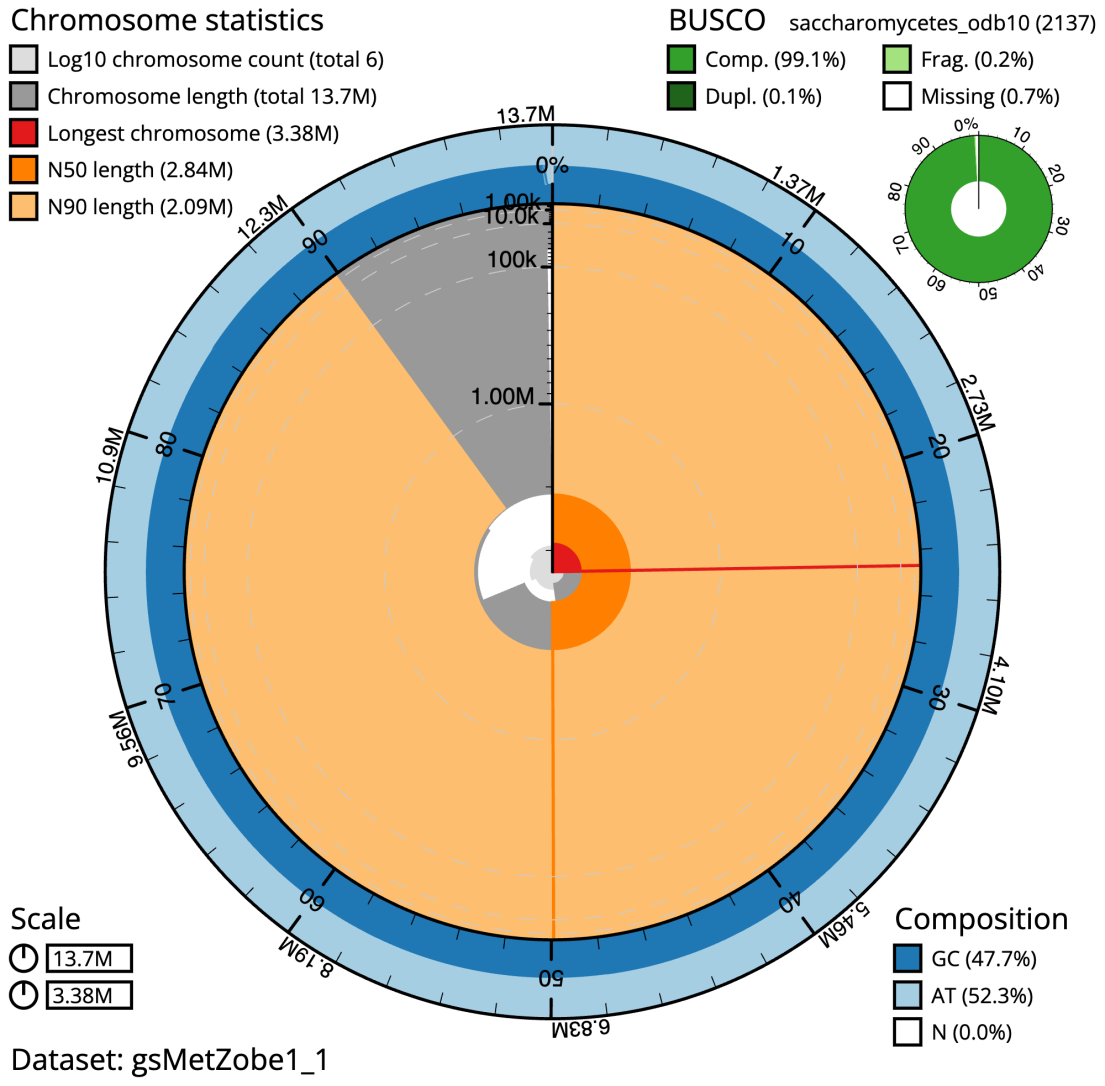
Genome assembly of *Metschnikowia zobellii*, gsMetZobe1.1: metrics. The BlobToolKit Snailplot shows N50 metrics and BUSCO gene completeness. The main plot is divided into 1,000 size-ordered bins around the circumference with each bin representing 0.1% of the 13,653,384 bp assembly. The distribution of scaffold lengths is shown in dark grey with the plot radius scaled to the longest scaffold present in the assembly (3,378,874 bp, shown in red). Orange and pale-orange arcs show the N50 and N90 scaffold lengths (2,836,740 and 2,093,737 bp), respectively. The pale grey spiral shows the cumulative scaffold count on a log scale with white scale lines showing successive orders of magnitude. The blue and pale-blue area around the outside of the plot shows the distribution of GC, AT and N percentages in the same bins as the inner plot. A summary of complete, fragmented, duplicated and missing BUSCO genes in the saccharomycetes_odb10 set is shown in the top right. An interactive version of this figure is available at https://blobtoolkit.genomehubs.org/view/gsMetZobe1.1/dataset/gsMetZobe1_1/snail.

The final assembly has a total length of 13.6 Mb in 5 sequence scaffolds with a scaffold N50 of 2.8 Mb ( Table 1). The whole assembly sequence was assigned to 5 chromosomal-level scaffolds. Chromosome-scale scaffolds confirmed by the Hi-C data are named in order of size ( Figure 1– Figure 4; Table 2). While not fully phased, the assembly deposited is of one haplotype. Contigs corresponding to the second haplotype have also been deposited. The mitochondrial genome was also assembled and can be found as a contig within the multifasta file of the genome submission.

**Table 1.** Genome data for *Metschnikowia zobellii*, gsMetZobe1.1.

Project accession data		
Assembly identifier	gsMetZobe1.1	
Species	*Metschnikowia zobellii*	
Specimen	gsMetZobe1	
NCBI taxonomy ID	27328	
BioProject	PRJEB52022	
BioSample ID	SAMEA7500987	
Isolate information	gsMetZobe1: (DNA sequencing, Hi-C scaffolding)	
Assembly metrics *		*Benchmark*
Consensus quality (QV)	69.7	*≥ 50*
*k*-mer completeness	100%	*≥ 95%*
BUSCO **	C:99.1%[S:98.9%,D:0.1%], F:0.2%,M:0.7%,n:2,137	*C ≥ 95%*
Percentage of assembly mapped to chromosomes	100%	*≥ 95%*
Sex chromosomes	-	*localised homologous pairs*
Organelles	Mitochondrial genome assembled	*complete single alleles*
Raw data accessions		
PacificBiosciences SEQUEL II	ERR9588940, ERR9588941	
Hi-C Illumina	ERR9503460	
Genome assembly		
Assembly accession	GCA_939531405.1	
*Accession of alternate haplotype*	GCA_939531315.1	
Span (Mb)	13.6	
Number of contigs	7	
Contig N50 length (Mb)	2.8	
Number of scaffolds	5	
Scaffold N50 length (Mb)	2.8	
Longest scaffold (Mb)	3.4	

* Assembly metric benchmarks are adapted from column VGP-2020 of “Table 1: Proposed standards and metrics for defining genome assembly quality” from ( Rhie *et al.*, 2021). ** BUSCO scores based on the saccharomycetes_odb10 BUSCO set using v5.3.2. C = complete [S = single copy, D = duplicated], F = fragmented, M = missing, n = number of orthologues in comparison. A full set of BUSCO scores is available at https://blobtoolkit.genomehubs.org/view/gsMetZobe1.1/dataset/gsMetZobe1_1/busco.

**Figure 2.**
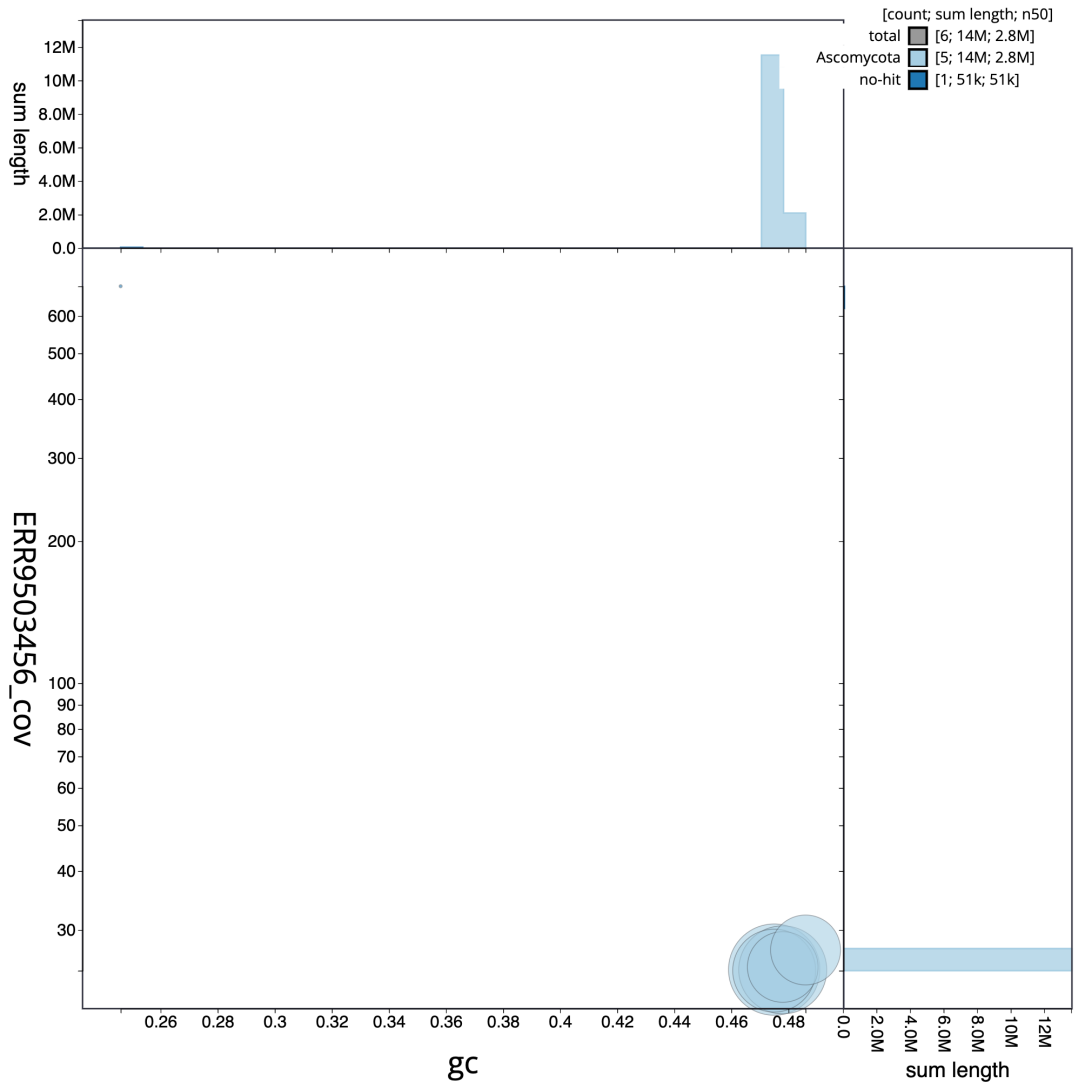
Genome assembly of *Metschnikowia zobellii*, gsMetZobe1.1: BlobToolKit GC-coverage plot. Scaffolds are coloured by phylum. Circles are sized in proportion to scaffold length. Histograms show the distribution of scaffold length sum along each axis. An interactive version of this figure is available at https://blobtoolkit.genomehubs.org/view/gsMetZobe1.1/dataset/gsMetZobe1_1/blob.

**Figure 3.**
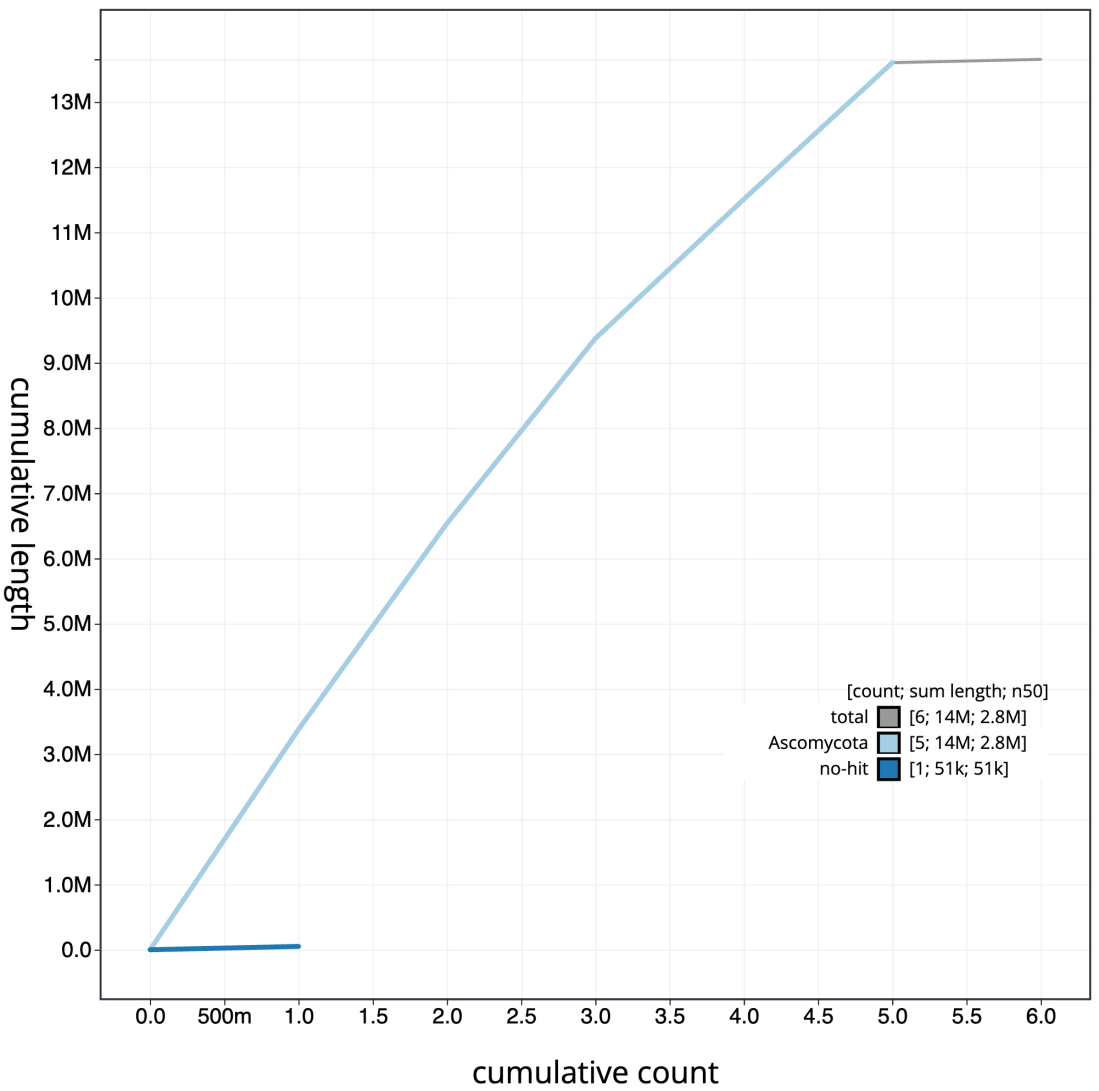
Genome assembly of *Metschnikowia zobellii*, gsMetZobe1.1: BlobToolKit cumulative sequence plot. The grey line shows cumulative length for all scaffolds. Coloured lines show cumulative lengths of scaffolds assigned to each phylum using the buscogenes taxrule. An interactive version of this figure is available at https://blobtoolkit.genomehubs.org/view/gsMetZobe1.1/dataset/gsMetZobe1_1/cumulative.

**Figure 4.**
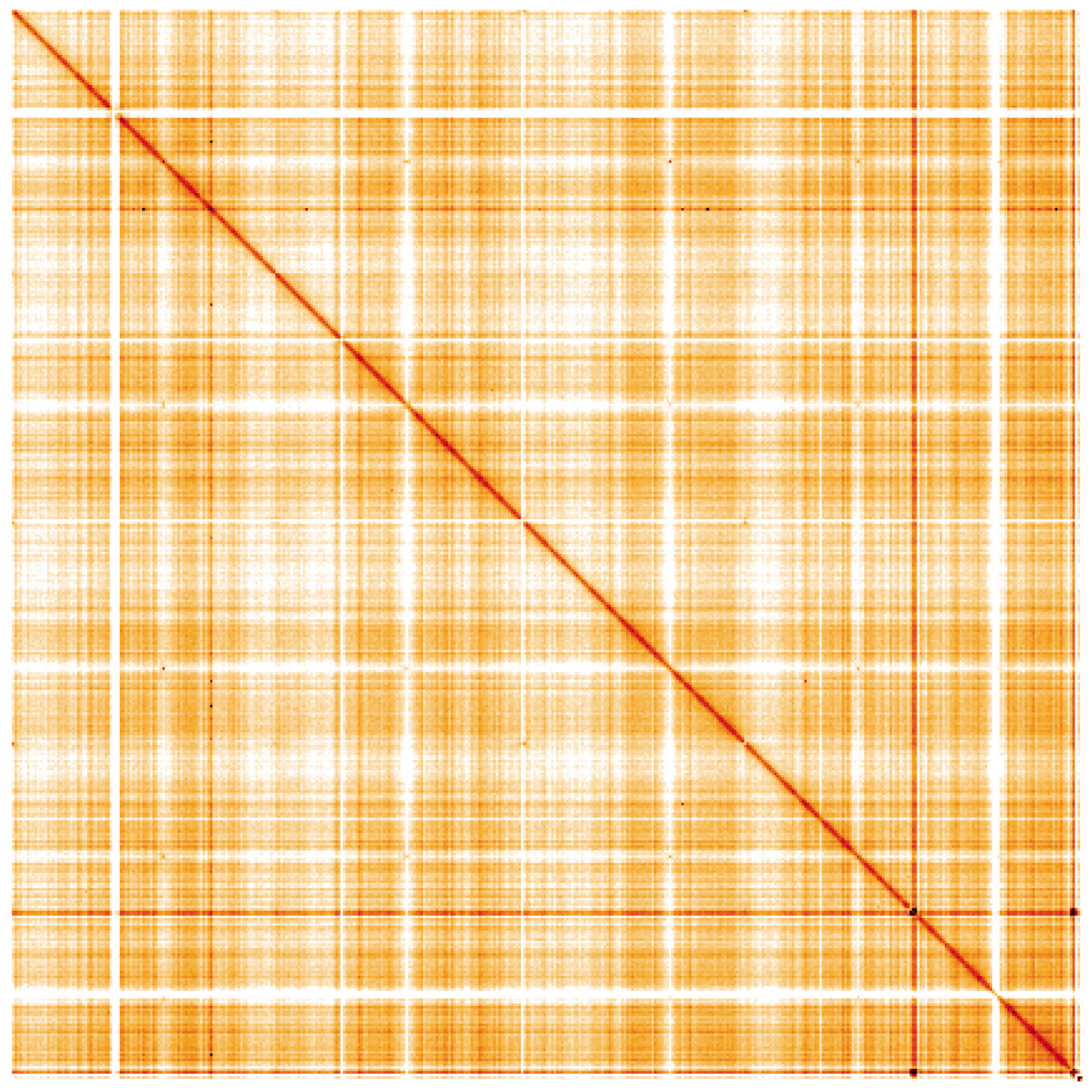
Genome assembly of *Metschnikowia zobellii*, gsMetZobe1.1: Hi-C contact map of the gsMetZobe1.1 assembly, visualised using HiGlass. Chromosomes are shown in order of size from left to right and top to bottom. An interactive version of this figure may be viewed at https://genome-note-higlass.tol.sanger.ac.uk/l/?d=MjYP2bWrTqimY9doy8ZPjA.

**Table 2.** Chromosomal pseudomolecules in the genome assembly of *Metschnikowia zobellii*, gsMetZobe1.

INSDC accession	Chromosome	Length (Mb)	GC%
OW618028.1	1	3.38	47.5
OW618029.1	2	3.16	48.0
OW618030.1	3	2.84	47.5
OW618031.1	4	2.13	48.0
OW618032.1	5	2.09	48.5
OW618033.1	MT	0.05	24.5

The estimated Quality Value (QV) of the final assembly is 69.7 with *k*-mer completeness of 100%, and the assembly has a BUSCO v5.3.2 completeness of 99.1% (single = 98.9%, duplicated = 0.1%), using the saccharomycetes_odb10 reference set ( *n* = 2,137).

Metadata for specimens, spectral estimates, sequencing runs, contaminants and pre-curation assembly statistics can be found at https://links.tol.sanger.ac.uk/species/27328.

## Methods

### Sample acquisition and nucleic acid extraction

A colony of *Metschnikowia zobellii* (specimen ID MBA-191008-001A, individual gsMetZobe1) was collected from Church Reef, Wembury, Devon, UK (latitude 50.32, longitude –4.08) on 2019-10-08. The specimen was collected by Michael Cunliffe (Marine Biological Association) and grown on agar. The identifier was Ro Allen (Marine Biological Association). The sample was harvested and preserved in liquid nitrogen before processing.

DNA was extracted at the Tree of Life laboratory, Wellcome Sanger Institute (WSI). The gsMetZobe1 sample was weighed and some of the sample was set aside for Hi-C sequencing. The cells were cryogenically disrupted to a fine powder using a Covaris cryoPREP Automated Dry Pulveriser, receiving multiple impacts. High molecular weight (HMW) DNA was extracted using the Qiagen Plant MagAttract v3 DNA extraction kit. HMW DNA was sheared into an average fragment size of 12–20 kb in a Megaruptor 3 system with speed setting 30. Sheared DNA was purified by solid-phase reversible immobilisation using AMPure PB beads with a 1.8X ratio of beads to sample to remove the shorter fragments and concentrate the DNA sample. The concentration of the sheared and purified DNA was assessed using a Nanodrop spectrophotometer and Qubit Fluorometer and Qubit dsDNA High Sensitivity Assay kit. Fragment size distribution was evaluated by running the sample on the FemtoPulse system.

### Sequencing

Pacific Biosciences HiFi circular consensus DNA sequencing libraries were constructed according to the manufacturers’ instructions. DNA sequencing was performed by the Scientific Operations core at the WSI on a Pacific Biosciences SEQUEL II (HiFi) instrument. Hi-C data were also generated from gsMetZobe1 using the Arima2 kit and sequenced on the Illumina NovaSeq 6000 instrument.

### Genome assembly, curation and evaluation

Assembly was carried out with Hifiasm ( Cheng *et al.*, 2021) and haplotypic duplication was identified and removed with purge_dups ( Guan *et al.*, 2020). The assembly was then scaffolded with Hi-C data ( Rao *et al.*, 2014) using SALSA2 ( Ghurye *et al.*, 2019). The assembly was checked for contamination and corrected as described previously ( Howe *et al.*, 2021). Manual curation was performed using HiGlass ( Kerpedjiev *et al.*, 2018) and Pretext ( Harry, 2022). The mitochondrial genome was assembled using MitoHiFi ( Uliano-Silva *et al.*, 2023), which runs MitoFinder ( Allio *et al.*, 2020) or MITOS ( Bernt *et al.*, 2013) and uses these annotations to select the final mitochondrial contig and to ensure the general quality of the sequence.

A Hi-C map for the final assembly was produced using bwa-mem2 ( Vasimuddin *et al.*, 2019) in the Cooler file format ( Abdennur & Mirny, 2020). To assess the assembly metrics, the *k*-mer completeness and QV consensus quality values were calculated in Merqury ( Rhie *et al.*, 2020). This work was done using Nextflow ( Di Tommaso *et al.*, 2017) DSL2 pipelines “sanger-tol/readmapping” ( Surana *et al.*, 2023a) and “sanger-tol/genomenote” ( Surana *et al.*, 2023b). The genome was analysed within the BlobToolKit environment ( Challis *et al.*, 2020) and BUSCO scores ( Manni *et al.*, 2021; Simão *et al.*, 2015) were calculated.

Table 3 contains a list of relevant software tool versions and sources.

**Table 3.** Software tools: versions and sources.

Software tool	Version	Source
BlobToolKit	3.4.0	https://github.com/blobtoolkit/blobtoolkit
BUSCO	5.3.2	https://gitlab.com/ezlab/busco
FreeBayes	1.3.1-17-gaa2ace8	https://github.com/freebayes/freebayes
gEVAL	N/A	https://geval.org.uk/
Hifiasm	0.16.1-r375	https://github.com/chhylp123/hifiasm
HiGlass	1.11.6	https://github.com/higlass/higlass
Long Ranger ALIGN	2.2.2	https://support.10xgenomics.com/genome-exome/ software/pipelines/latest/advanced/other-pipelines
Merqury	MerquryFK	https://github.com/thegenemyers/MERQURY.FK
MitoHiFi	2	https://github.com/marcelauliano/MitoHiFi
PretextView	0.2	https://github.com/wtsi-hpag/PretextView
purge_dups	1.2.3	https://github.com/dfguan/purge_dups
SALSA	2.3	https://github.com/salsa-rs/salsa
sanger-tol/genomenote	v1.0	https://github.com/sanger-tol/genomenote
sanger-tol/readmapping	1.1.0	https://github.com/sanger-tol/readmapping/tree/1.1.0

### Wellcome Sanger Institute – Legal and Governance

The materials that have contributed to this genome note have been supplied by a Darwin Tree of Life Partner. The submission of materials by a Darwin Tree of Life Partner is subject to the **‘Darwin Tree of Life Project Sampling Code of Practice’**, which can be found in full on the Darwin Tree of Life website here. By agreeing with and signing up to the Sampling Code of Practice, the Darwin Tree of Life Partner agrees they will meet the legal and ethical requirements and standards set out within this document in respect of all samples acquired for, and supplied to, the Darwin Tree of Life Project.

Further, the Wellcome Sanger Institute employs a process whereby due diligence is carried out proportionate to the nature of the materials themselves, and the circumstances under which they have been/are to be collected and provided for use. The purpose of this is to address and mitigate any potential legal and/or ethical implications of receipt and use of the materials as part of the research project, and to ensure that in doing so we align with best practice wherever possible. The overarching areas of consideration are:

• Ethical review of provenance and sourcing of the material

• Legality of collection, transfer and use (national and international) 

Each transfer of samples is further undertaken according to a Research Collaboration Agreement or Material Transfer Agreement entered into by the Darwin Tree of Life Partner, Genome Research Limited (operating as the Wellcome Sanger Institute), and in some circumstances other Darwin Tree of Life collaborators.

## Data Availability

European Nucleotide Archive: *Metschnikowia zobellii* (a marine yeast). Accession number PRJEB52022; https://identifiers.org/ena.embl/PRJEB52022. ( Wellcome Sanger Institute, 2022) The genome sequence is released openly for reuse. The *Metschnikowia zobellii* genome sequencing initiative is part of the Darwin Tree of Life (DToL) project. All raw sequence data and the assembly have been deposited in INSDC databases. The genome will be annotated using available RNA-Seq data and presented through the Ensembl pipeline at the European Bioinformatics Institute. Raw data and assembly accession identifiers are reported in Table 1.

## Author information

Members of the Marine Biological Association Genome Acquisition Lab are listed here: https://doi.org/10.5281/zenodo.4783604.

Members of the Darwin Tree of Life Barcoding collective are listed here: https://doi.org/10.5281/zenodo.4893703.

Members of the Wellcome Sanger Institute Tree of Life programme are listed here: https://doi.org/10.5281/zenodo.4783585.

Members of Wellcome Sanger Institute Scientific Operations: DNA Pipelines collective are listed here: https://doi.org/10.5281/zenodo.4790455.

Members of the Tree of Life Core Informatics collective are listed here: https://doi.org/10.5281/zenodo.5013541.

Members of the Darwin Tree of Life Consortium are listed here: https://doi.org/10.5281/zenodo.4783558.
