# Supplementary material for: The genome sequence of a marine yeast, Metschnikowia zobellii (Uden & Cast.-Branco, 1961)
Source: Wellcome Open Res. 2023 Sep 19;8:411. [Version 1] doi: 10.12688/wellcomeopenres.19998.1 (PMC11322703; doi:10.12688/wellcomeopenres.19998.1)
Supplement: Peer review history

# Reviewer report: Reviewer response for version 1

**DOI:** 10.21956/wellcomeopenres.22144.r89205

**Published:** 2025-03-18

**Authors:** Richard Edwards

**Affiliations:** 1 Oceans Institute, The University of Western Australia (Ringgold ID: 590735), Crawley, Western Australia, Australia; 2 School of Biotechnology and Biomolecular Sciences, University of New South Wales, Sydney, New South Wales, Australia

This manuscript describes a near-gap-free haploid genome assembly of a marine yeast species. The methods and data are well described and appear to be of excellent quality. The assembly consists of six sequences: five nuclear chromosomes and a mitochondrial genome. There is a little confusion with the reporting of this: Figure 2 clearly has six scaffolds but Table 1 only reports five. Please make this consistent. It would also be helpful if the chromosomes were clearly marked in Figure 4.

 Table 1 reports seven contigs, which means that at least three of the chromosomes are gap-free. Are the chromosomes all terminated with telomeres at each end? If so, this should be reported as a telomere-to-telomere (T2T) assembly. Either way, I think it is important and useful to report the telomere status in Table 2. Given that there are only one or two gaps in the whole assembly, it would be interesting to know where these are. 

 Finally, was the genome size estimated? The original *Saccharomyces cerevisiae *genome had almost 1 Mb missing due to collapsed rDNA repeats. *Metschnikowia zobellii* is a similarly small genome, and it would be useful if something similar is the case here. If the assembly is not T2T (see above) then there might also be some sequence missing at the ends of the chromosomes.

 Neither of these analyses are essential, but if the assembly is essentially a gap-free T2T genome then it is worth drawing attention to this fact as it increases the future utility of the assembly.

Are sufficient details of methods and materials provided to allow replication by others?

Yes

Is the rationale for creating the dataset(s) clearly described?

Yes

Are the datasets clearly presented in a useable and accessible format?

Yes

Are the protocols appropriate and is the work technically sound?

Yes

Reviewer Expertise:

Biodiversity genomics, genome assembly, bioinformatics, molecular evolution

I confirm that I have read this submission and believe that I have an appropriate level of expertise to confirm that it is of an acceptable scientific standard.

# Reviewer report: Reviewer response for version 1

**DOI:** 10.21956/wellcomeopenres.22144.r90278

**Published:** 2024-08-14

**Authors:** Suriana Sabri

**Affiliations:** 1 Universiti Putra Malaysia, Selangor, Malaysia

The paper presents a detailed genome assembly of the marine yeast *Metschnikowia zobellii*. This work is a significant contribution to the field, providing one of the first marine fungal genomes, which is crucial for advancing our understanding of the biology and ecology of marine fungi, particularly in the context of host-pathogen interactions. The assembly of *M. zobellii* is of high quality, spanning 13.6 Mb and organized into five chromosomal pseudomolecules. The use of advanced sequencing technologies, including Pacific Biosciences HiFi and Hi-C data, has resulted in a robust and comprehensive genome assembly. The inclusion of the mitochondrial genome adds further value to the study. This genome provides a foundational resource for marine fungal studies, with significant implications for understanding marine ecosystems and the dynamics of plankton populations, where *M. zobellii* plays a role. 

 The paper is well-executed, methodologically sound, and provides important insights into marine fungal genomics. Therefore, it should be accepted for indexing.

Are sufficient details of methods and materials provided to allow replication by others?

Yes

Is the rationale for creating the dataset(s) clearly described?

Yes

Are the datasets clearly presented in a useable and accessible format?

Yes

Are the protocols appropriate and is the work technically sound?

Yes

Reviewer Expertise:

Microbial genomics

I confirm that I have read this submission and believe that I have an appropriate level of expertise to confirm that it is of an acceptable scientific standard.

# Reviewer report: Reviewer response for version 1

**DOI:** 10.21956/wellcomeopenres.22144.r90273

**Published:** 2024-08-13

**Authors:** Yi Wu

**Affiliations:** 1 Tianjin University, Tianjin, China

The authors have provided the complete genome sequence of *Metschnikowia zobellii* in this study. This will provide data resources for multiple biology fields. I noticed that *Metschnikowia zobellii* has less than 1% genomic difference from *Saccharomyces cerevisiae*, which makes me curious about how this portion of the genome results in the substantial differences in the survival strategies of the two yeasts.

 Overall, the paper presents comprehensive data with clear and intuitive analysis. Here are my comments:

1. **Introduction**: The introduction provides a good overview of the research background and objectives. However, it could further discuss the specific role of *Metschnikowia zobellii *in marine ecosystems and its biotechnological potential.
2. **Methods**: The methods are described in detail. However, if the authors could provide more comprehensive annotation of the assembled genome, it would greatly benefit related research fields.

Are sufficient details of methods and materials provided to allow replication by others?

Yes

Is the rationale for creating the dataset(s) clearly described?

Yes

Are the datasets clearly presented in a useable and accessible format?

Yes

Are the protocols appropriate and is the work technically sound?

Yes

Reviewer Expertise:

Yeast, synthetic biology

I confirm that I have read this submission and believe that I have an appropriate level of expertise to confirm that it is of an acceptable scientific standard.

# Reviewer report: Reviewer response for version 1

**DOI:** 10.21956/wellcomeopenres.22144.r90279

**Published:** 2024-08-05

**Authors:** Marc-André Lachance

**Affiliations:** 1 University of Western Ontario, Ontario, Canada

A high definition sequence of *Metschnikowia zobellii* is presented.

 The following are comments that might help improve the final version of the article:

- In the background paragraph, a reference to Seki and Fulton 1969 is cited in support of a statement regarding *Metschnikowia zobellii*. Examination of the reference reveals that the species was identified as *M. krissii*. Having said this, the identification tools available in 1969 (and the data in the 1969 paper) would suggest that a great deal of prudence should be exercised in reporting identifications to the species level performed in the pre-sequencing era.
- Secondly, a statement is made, referring to Chrismas *et al.*, 2023, on the occurrence of *Metschnikowia *in nature. Care should be exercised here, as the said reference does not specify what species of *Metschnikowia *exhibits annual recurrence. The genus *Metschnikowia *is large and diverse in the ecology of its species, such that the reference may not pertain to *M. zobellii* (although it might, but we don’t know).
- Additional information on the strain would be useful, namely the collection where the strain was deposited and the accession number.  I presume the strain is publicly available. Identification is attributed to an individual, but the method used and the supporting data (D1/D2 sequence) should be specified. I could not find a relevant GenBank deposit. A D1/D2 sequence would be essential and is not always extractable from whole genomes.
- I do not see any mention of an already existing genome sequence for *M. zobellii*, although there is one: GCA_030572635.1. The sequence has been used in a large phylogeny of hundreds of ascomycetous yeasts (Opulente *et al*., 2024) ^1^. It might be useful to give a short comparison of the two datasets.

Are sufficient details of methods and materials provided to allow replication by others?

Yes

Is the rationale for creating the dataset(s) clearly described?

Partly

Are the datasets clearly presented in a useable and accessible format?

Yes

Are the protocols appropriate and is the work technically sound?

Yes

Reviewer Expertise:

Systematics, ecology, and evolution of yeasts, in particular ascomycetous species, including Metschnikowia. I am not an expert on genome assembly and annotation software.

I confirm that I have read this submission and believe that I have an appropriate level of expertise to confirm that it is of an acceptable scientific standard, however I have significant reservations, as outlined above.

## References

[1] : Genomic factors shape carbon and nitrogen metabolic niche breadth across Saccharomycotina yeasts. *Science* .2024;384(6694) : 10.1126/science.adj4503 eadj4503 10.1126/science.adj4503 38662846 PMC11298794

# Reviewer report: Reviewer response for version 1

**DOI:** 10.21956/wellcomeopenres.22144.r87867

**Published:** 2024-07-25

**Authors:** Soo-Je Park

**Affiliations:** 1 Department of Biology, Jeju National University, Jeju-si, Jeju-do, South Korea

In this study, the authors described a high-completeness (i.e., high-qulity) genome of a marine yeast, *Metschnikowia zobellii* as a pathogen for *Daphnia magna*. The genome sequences were generated by HiFi technique. The genome is reported to be 13.6 Mb and comprised of 5 scaffolds. Also, the mitochondrial genome was assembled into 51.1 Kb. Protocols used were clearly presented and all data is publicly available within ENA (project: PRJEB52022).

 Minor comments:

- For the reader, the authors should be provided with protocols for specific purposes.
- It is requested that the specific goals for the analysis tools used be defined, rather than a general statement such as "The genome was analyzed in the BlobToolKit environment." For example, Busco was used to estimate genome completeness, and Hifiasm was used for de novo assembly. This information should be included in Table 3.

Are sufficient details of methods and materials provided to allow replication by others?

Yes

Is the rationale for creating the dataset(s) clearly described?

Yes

Are the datasets clearly presented in a useable and accessible format?

Yes

Are the protocols appropriate and is the work technically sound?

Yes

Reviewer Expertise:

environmental genomics, environmental microbiology, ecogenomics.

I confirm that I have read this submission and believe that I have an appropriate level of expertise to confirm that it is of an acceptable scientific standard.
